# Medication use in Poland for children and adolescents with neurodevelopmental disorders: before, during and after the COVID-19 pandemic - a retrospective pharmacoepidemiological study using national reimbursement data

**DOI:** 10.3389/fphar.2025.1728388

**Published:** 2026-01-07

**Authors:** Andrzej Silczuk, Malwina Hołownia-Voloskova, Anna Mosiołek, Otton Roubinek, Katarzyna Bliźniewska-Kowalska, Krzysztof Marcin Zakrzewski, Marcin Czech

**Affiliations:** 1 Department of Community Psychiatry, Faculty of Life Sciences, Medical University of Warsaw, Warsaw, Poland; 2 Department of Pharmacoeconomics, Institute of Mother and Child, Warsaw, Poland; 3 Department of Interdisciplinary Disability Studies, The Maria Grzegorzewska University of Special Education, Warsaw, Poland; 4 Pharmacy and Biotechnology Center, Lukasiewicz Research Network - Industrial Chemistry Institute, Warsaw, Poland; 5 Department of Adult Psychiatry, Medical University of Lodz, Lodz, Poland; 6 The Independent Group of Public Ambulatory Care Institutions Warsaw-Ochota, Warsaw, Poland

**Keywords:** ADHD, COVID-19 pandemic, medication dispensing, neurodevelopmental disorders, Pharmacoepidemiology, pharmacotherapy 3

## Abstract

**Background:**

The pandemic profoundly disrupted healthcare and education systems, potentially affecting the pharmacological management of neurodevelopmental disorders (NDDs) in children and adolescents.

**Objective:**

To examine national trends in psychotropic drug dispensing among pediatric patients with NDDs in Poland before, during, and after the COVID-19 pandemic.

**Methods:**

A retrospective pharmacoepidemiological analysis was conducted using data from the IQVIA Pharmascope database, which records all reimbursed medicines dispensed by community pharmacies in Poland. Monthly and annual dispensing volumes were analyzed for January 2018–December 2024, focusing on drugs commonly used in ADHD, autism spectrum disorder, and intellectual disability. Three time periods were compared: pre-pandemic (2018–February 2020), pandemic (March 2020–June 2022), and post-pandemic (July 2022–December 2024).

**Results:**

A marked surge in psychotropic drug dispensing was observed in 2021, with total annual utilization jumping to nearly 2.7 billion dispensed days of therapy (DOT)—more than double the levels seen in any prior year. This represents a 161% increase compared with 2020, when volumes were approximately 1.0 billion DOT. The most substantial increases occurred for sedative and anxiolytic agents (hydroxyzine, diazepam, alprazolam, lorazepam) and antipsychotics (olanzapine, aripiprazole, risperidone, chlorprothixene, haloperidol, levomepromazine), consistent with pandemic related stress, limited access to non-pharmacological care, and possible stockpiling. Dispensing volumes fell sharply in 2022 to around 1.1 billion DOT, returning close to pre-pandemic levels and suggesting these effects were transient. A modest upward trend resumed thereafter, with volumes rising to approximately 1.2 billion DOT in 2023 and 1.5 billion DOT in 2024, indicating gradual recovery but remaining far below the extraordinary 2021 peak.

**Conclusion:**

The COVID-19 pandemic induced broad but temporary increases in pediatric psychotropic drug dispensing, except for ADHD pharmacotherapies, which demonstrated a persistent upward trend. These findings suggest lasting shifts in diagnostic and therapeutic practices and underscore the need for continued monitoring of stimulant use and prescribing appropriateness in pediatric neurodevelopmental care.

## Introduction

Neurodevelopmental disorders are conditions with onset in early childhood that involve intellectual, social, communication, and behavioral deficits (Thapar et al., 2017; Johnson et al., 2015) and include autism spectrum disorder, intellectual developmental disorders, attention deficit hyperactivity disorder, and related conditions that substantially affect educational attainment, social integration, and quality of life for affected children and their families (Baxter et al., 2015; Tick et al., 2016).

Neurodevelopmental disorders (NDDs) such as autism spectrum disorder (ASD), attention-deficit/hyperactivity disorder (ADHD), intellectual disability, and communication disorders (i.e., childhood-onset fluency disorders (i.e., stuttering), language disorder, and social (pragmatic) communication disorder) are a group of heterogeneous conditions whose symptoms manifest in early childhood. Their etiology is complex, with many genetic predispositions, prenatal and perinatal factors, as well as environmental exposures, which interfere with brain development (Thapar et al., 2017; Johnson et al., 2015; Baxter et al., 2015).

### Genetic factors

Genetic predispositions are also critical underlying factors of NDDs. Family and twin studies have consistently revealed a high heritability, especially for ASD (50%–90%) and ADHD (70%–80%) (Tick et al., 2016; Faraone and Larsson, 2019). More than 100 rare single-gene disorders and chromosomal disorders have been associated with these disorders, such as fragile X syndrome, Rett syndrome, and 22q11.2 deletion syndrome (Abrahams and Geschwind, 2008; Vorstman et al., 2017). In addition, there is a contribution of polygenic risk, i.e., the impact of many small-effect common variants which interact with environmental factors to modulate phenotypic expression (Grove et al., 2019).

### Prenatal risk factors

Disturbances in fetal neurodevelopment can be due to maternal infections, immune stimulus, metabolic disorders, insufficient nutrients, or teratogenic agents (Brown and Patterson, 2011; Ornoy et al., 2015). In terms of infections, maternal rubella, cytomegalovirus, and toxoplasmosis, as well as maternal influenza, are all related to higher NDD risk (Atladóttir et al., 2010), whereas diabetes, obesity, thyroid disorder, and autoimmune diseases have been related to increased prevalence of ASD and ADHD among offspring (Krakowiak et al., 2012; Brown and Susser, 2008). Other nutritional factors such as folate and vitamin D deficiencies, as well as low omega-3 polyunsaturated fatty acid intake during pregnancy, have also been implicated in aberrant structural and functional brain development (Vuillermot et al., 2017). Last, prenatal exposures to alcohol (fetal alcohol spectrum disorders), tobacco, and drugs lead to structural and functional brain changes that result in cognitive and behavioral difficulties (Mattson et al., 2019).

### Perinatal and birth-related factors

Perinatal factors include preterm birth, low birth weight, hypoxic–ischemic encephalopathy, and severe neonatal jaundice; all of which are risk factors for NDDs (John et al., 2017; Odd et al., 2008). Perinatal brain injury at critical neurodevelopmental intervals may disturb neuronal migration, synaptogenesis, and myelination (Volpe, 2009).

### Postnatal environmental and social factors

Early-life exposures shape brain development across the life course. Lead, mercury, and organophosphate pesticide toxins are linked to deficits in behavior and cognition (Bel and linger, 2008; Shelton et al., 2012). Profound early psychosocial deprivation like what we see in institutionalized children has been associated with decreased cortical volume, poor executive function, and elevated ASD symptoms (Tottenham, 2014; Rutter et al., 2010).

The etiological panorama of NDDs is complex and involves a combination of genetic susceptibility and environmental insults during prenatal, perinatal, and postnatal development. Such risk factors are largely modifiable factors, suggesting the potential to prevent them from occurring by improving maternal health and early diagnosis and through the reduction of harmful exposures.

The COVID-19 pandemic, as declared by the World Health Organization in March 2020 (Cucinotta and Vanelli, 2020), has led to unprecedented disruptions to healthcare, education, and daily life. In Poland, the first national lockdown started in March 2020, during which school had been closed, non-urgent medical services were suspended, and in-person consultations had been limited. Successive surges of the virus resulted in a series of partial and full lockdowns, and restrictions on movement remained in place throughout much of 2021 (European Centre for Disease Prevention and Control, 2021; Poland, 2021). Unintended consequences of these actions, taken to reduce infection spread, resulted in a lose-lose-lose situation for children with NDDs and their families. With reduced private access to specialist visits, therapy appointments, and in-school support, and pandemic stress, disruptions of routine, and social isolation, these made behavioral, emotional challenges and symptoms worse for many children (Colizzi et al., 2020; Patel, 2020).

Evidence from Europe and elsewhere, suggests that during the pandemic, prescribing of psychotropic drugs in children and adolescents increased, especially regarding sedatives, anxiolytics, and ADHD drugs (Yanga et al., 2020; Pelinovskya et al., 2022). Putative contributing factors involve increased anxiety and sleep problems, increased behavioral dysregulation, and more reliance on pharmacological approaches when access to non-pharmacologic based interventions was restricted (Taquet et al., 2021). In addition, short term stockpiling at the level of pharmacies or caregivers may have produced brief, local spikes in dispensing volumes around periods of supply disruption or anticipated access limitations, although such behavior would not be expected to influence long term prescribing trends (Patel et al., 2020).

This study analyzes national level dispensing patterns of selected psychotropic medications used in children and adolescents in Poland before, during, and after the COVID-19 pandemic. The medications were chosen based on their established clinical role in the management of neurodevelopmental disorders in pediatric populations and their inclusion in the national reimbursement system. In Poland, reimbursed medications are partially or fully covered by the National Health Fund, which means that patients pay only a regulated copayment and do not receive reimbursement retroactively; therefore, the dataset captures only subsidized outpatient prescriptions and excludes private or fully out of pocket purchases. Drugs with minimal relevance to chronic neurodevelopmental care, those rarely prescribed in pediatric practice, or those not reimbursed were excluded because they are not reliably represented in the available data. Monthly dispensing volumes from January 2018 to December 2024 were obtained from the IQVIA Pharmascope database, which records all reimbursed medications dispensed by community pharmacies nationwide. Although the dataset does not distinguish between indications, most of the medications included are routinely used in neurodevelopmental disorder management. The aim of the analysis was to identify which medication classes showed temporary *versus* sustained shifts in dispensing across the pandemic phases and to consider how these patterns may reflect emerging long-term changes in psychotropic prescribing for children and adolescents with neurodevelopmental conditions.

## Materials and methods

### Study design and setting

This was a retrospective study analyzing national-level pharmaceutical reimbursement data to assess trends in the use of selected psychotropic medications among children diagnosed with selected neurodevelopmental disorders in Poland. It is important to note that reimbursed prescription data in this source are not linked to individual diagnostic codes at the patient level. The analytic assumption that these medications are used predominantly within neurodevelopmental disorders reflects established clinical practice, but indication specific attribution cannot be confirmed in this dataset.

Although the study uses the COVID-19 pandemic as a temporal reference, the methodological framework based on longitudinal national reimbursement data, predefined time strata, and class specific dispensing trends is generalizable. It can be applied to monitor system level shifts in prescribing associated with policy changes, healthcare access modifications, or other external events. The pandemic therefore serves as a natural experiment illustrating how this analytical structure captures abrupt and sustained changes in psychotropic medication use.

### Data source

Prescription purchase data were obtained from the IQVIA Pharmascope database, which captures all reimbursed medicinal products dispensed in community pharmacies in Poland. The dataset provides monthly aggregated sales volumes, expressed in standard units, for each reimbursed product and is representative at the national level. Because the dataset is fully anonymized and aggregated, it does not include diagnostic indications, patient characteristics, or treatment duration; therefore, the analysis describes dispensing patterns rather than diagnosis-based prescribing. As the study used anonymized national reimbursement data without any patient-level identifiers, ethics committee approval and informed consent were not required. In the Polish healthcare system, diagnostic codes are not linked to individual prescriptions in pharmacy reimbursement datasets. Although these medications are commonly prescribed for neurodevelopmental disorders, the IQVIA Pharmascope data do not contain patient level ICD codes, and indication specific use cannot be confirmed. Therefore, the analysis reflects overall dispensing patterns rather than diagnosis verified prescribing. Additionally, age was defined according to the Polish healthcare system classification, where individuals under 18 years are considered pediatric patients for reimbursement purposes.

### Study period

Data were extracted for the period from January 2018 to December 2024, allowing for the comparison of prescription trends in three timeframes: pre-pandemic (January 2018–February 2020), pandemic (March 2020–June 2022), and post-pandemic (July 2022–December 2024).

### Population and diagnostic codes

The medications analyzed are prescribed, though not exclusively, in the context of the following ICD 10 diagnostic codes (World Health Organization, 1992). These diagnostic categories are presented using ICD 10 terminology because it is the official coding standard in the Polish healthcare and reimbursement system and corresponds to the framework used in the IQVIA dataset.

- Attention-deficit/hyperactivity disorder (ADHD): F90 – Hyperkinetic disorders, F90.0 – Disturbance of activity and attention (combined type ADHD), and F90.1 – Hyperkinetic conduct disorder.
- Childhood autism (F84.0), atypical autism (F84.1), Asperger’s syndrome (F84.5), other pervasive developmental disorders (F84.8).
- Intellectual disabilities: mild intellectual disability (F70), moderate intellectual disability (F71), severe intellectual disability (F72), and profound intellectual disability (F73).

These diagnostic categories are presented to contextualize the typical clinical use of the analyzed agents; however, the reimbursement dataset does not include patient level diagnostic codes. Therefore, the study cannot identify whether dispensed units were prescribed specifically for the neurodevelopmental indications listed above.

### Medications analyzed

For ADHD, the following active substances were included:

- Stimulant and non-stimulant medications: methylphenidate, atomoxetine, clonidine.
- Antipsychotics and others: aripiprazole, risperidone, chlorprothixene hydrochloride.

For other neurodevelopmental disorders (ASD, intellectual disabilities), the substances included were:

- Antipsychotics: aripiprazole, risperidone, haloperidol, olanzapine, chlorprothixene hydrochloride, levomepromazine.
- Benzodiazepines: diazepam, alprazolam, lorazepam, clonazepam.
- Other anxiolytics: hydroxyzine.

Other medication classes such as selective serotonin reuptake inhibitors, buspirone, sedating antihistamines including promethazine and chlorpheniramine, and sleep agents such as melatonin or zopiclone were not included because they are either not reimbursed for pediatric use, inconsistently captured in the national reimbursement system, or prescribed mainly for comorbid conditions rather than core neurodevelopmental disorder management. Therefore, they do not appear reliably in the dataset and fall outside the primary scope of this study.

### Data analysis

Monthly prescription volumes for each active substance were aggregated separately for the ADHD group and for the combined “other neurodevelopmental disorders” group. Used units were dispensed Rx (DOT), that represents the Days of Therapy (DOT) dispensed to patients. Days of therapy (DOT) is a measure defining the number of therapy days (according to Defined Daily Dosages, DDD) ensured by the number of packages of a given medication sold in each period. It is assumed that Defined Daily Dosages (DDD) correspond to the WHO guidelines defining the recommended daily dosage for each therapeutic substance. DOT is calculated by converting dispensed prescriptions (Rx) into the corresponding number of treatment days, based on unit strength (dose), dosing regimen, and pack size.

Results were summarized descriptively and compared between the three timeframes (pre-pandemic, pandemic, post-pandemic). Trends over time were evaluated visually using line graphs and numerically using percentage change calculations. Statistical analyses, where applicable, were performed. Because indication specific and patient level data were not available, the analysis was limited to descriptive methods. Inferential statistical approaches such as interrupted time series modelling, adjustment for seasonality, or causal modelling were not feasible within the constraints of the dataset. The objective of the analysis was therefore to identify population level temporal patterns in dispensing, rather than to establish causal associations with pandemic related changes.

## Results

**FIGURE 1:**
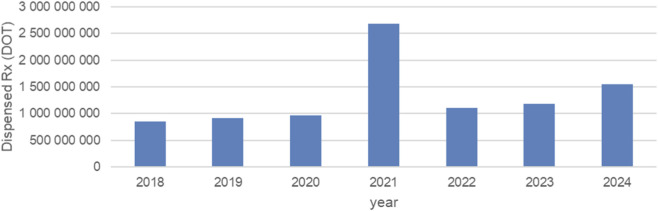
Neurodevelopmental and pervasive disorders drugs use in children and adolescents in Poland by years. Axis y: numbers of units, axis x: years of analysis.

Figure 1 displays the annual volume of dispensed days of therapy (DOT) for all neurodevelopmental-related medications combined between 2018 and 2024. From 2018 to 2020, annual utilization increased gradually from approximately 850 million DOT in 2018 to around 1.0 billion DOT in 2020, reflecting steady year-on-year growth in prescribing before the COVID-19 pandemic. In 2021, a marked and atypical surge occurred, with total annual dispensing rising sharply to nearly 2.7 billion DOT—more than double the utilization observed in any preceding year. This increase corresponds to the synchronous utilization spikes documented across most individual medications in the dataset during the early pandemic period. Following this peak, total dispensing dropped substantially in 2022 to around 1.1 billion DOT, returning close to pre-pandemic levels. A modest upward trajectory resumed thereafter, with volumes rising to approximately 1.2 billion DOT in 2023 and to around 1.5 billion DOT in 2024, indicating a gradual recovery but remaining well below the extraordinary 2021 levels. Overall, the figure highlights two dominant patterns: (1) a stable pre-pandemic baseline with modest growth, and (2) a pronounced, short-lived utilization spike in 2021 followed by a return to more typical prescribing patterns in subsequent years.

Between January 2018 and December 2024, total monthly dispensing volumes of the analyzed medicines remained relatively stable, except for a pronounced surge between January and August 2021 (Figure 1). This period coincided with the most restrictive phase of the COVID-19 pandemic in Poland, including the third national lockdown. Total monthly units for all products combined reached record highs during this interval before rapidly returning to baseline in late 2021.

The medicines included in this analysis are used for a range of conditions; the observed patterns therefore reflect all-indication utilization. However, many of these products are relevant for the pharmacological management of neurodevelopmental disorders (NDD), including autism spectrum disorder (ASD), intellectual disabilities, and ADHD.

### Class- and molecule-specific patterns

In line with the data presented in Table 1, sedating and anxiolytic agents showed the most pronounced pandemic-related surges. Hydroxyzine use rose from a baseline of approximately 0.45–0.50 million units per month to peaks of 1.4–1.6 million per month (around a threefold increase), while diazepam volumes increased from 0.14 to 0.17 million to 0.43–0.45 million units per month (2.5–3×). Alprazolam dispensing rose from 0.21 to 0.24 million to 0.68–0.71 million units per month (around threefold), and lorazepam volumes increased from 0.11 to 0.13 million to 0.30–0.32 million units per month (2.5–3×). For all four agents, these peaks occurred between January and August 2021 and were followed by a return to pre-pandemic levels by late 2021, with gradual downward trends observed in subsequent years.

**TABLE 1:** Approximate monthly dispensing volumes (all indications) for selected medicines, pre-pandemic baseline vs. 2021 peak, and post-pandemic trend.

Drug	Baseline (≈/mo)	2021 peak (≈/mo)	Peak vs. baseline	Post-2021 trend
Chlorprothixene	0.30–0.43 M	1.1–1.2 M	∼3–4×	Brief dip, then return to baseline
Diazepam	1.3–1.5 M	3.5–4.0 M	∼2.5–3×	↓ to ∼1.1–1.3 M
Lorazepam	2.0–2.4 M	5.5–6.5 M	∼2.5–3×	↓ to 1.6–2.0 M
Alprazolam	3.2–3.8 M	10–12 M	∼3×	Return to 3.0–3.6 M
Hydroxyzine	3.0–3.6 M	10–12 M	∼3–4×	Return to baseline
Aripiprazole	1.4–1.9 M	5.0–5.5 M	∼3×	Return to baseline (∼1.8–2.2 M)
Atomoxetine	20–50 k	180–250 k	∼4–6×	↑ steady rise to 150–200 k
Risperidone	1.2–1.6 M	3.5–4.0 M	∼2.5–3×	Return to baseline (∼1.3–1.6 M)
Haloperidol	0.80–1.05 M	2.2–2.6 M	∼2.5–3×	↓ to 0.8–0.9 M
Methylphenidate	0.3–0.7 M	2.2–2.6 M	∼3–4×	↑ strong growth to 2.0–3.0 M
Olanzapine	4.5–6.0 M	12–14 M	∼2.5–3×	Return to baseline (∼5–5.5 M)
Clonidine	50–70 M	180–200 M	∼3–4×	Return to baseline (∼70–90 M)
Levomepromazine	0.23–0.32 M	0.75–0.85 M	∼3×	Return to baseline
Clonazepam	1.8–2.2 M	5.5–6.0 M	∼2.5–3×	↓ to 1.5–1.8 M

M = million units; k = thousand units. Figures rounded from monthly averages in shared IQVIA data.

Antipsychotics frequently used in the management of neurodevelopmental disorders also demonstrated sharp but short-lived increases in 2021, with volumes normalizing thereafter. Olanzapine dispensing rose from 17 to 21 million to more than 55–60 million units per month (>2.5×), aripiprazole from 45 to 55 thousand to 120–140 thousand units per month (2.5–3×), and risperidone from 120 to 160 thousand to 350–380 thousand units per month (2.5–3×). Chlorprothixene increased from 70 to 90 thousand to 220–240 thousand units per month (threefold), with a brief dip in 2022 before returning to baseline, while haloperidol rose from 70 to 95 thousand to 200–220 thousand units per month (2.5–3×) and then declined slightly after 2021. Levomepromazine dispensing increased from 60 to 80 thousand to 190–200 thousand units per month (threefold).

In contrast, ADHD core therapies demonstrated sustained growth after the pandemic. Methylphenidate volumes rose from 10 to 20 thousand units per month pre-pandemic to a short-lived peak of around 80 thousand units per month in 2021, followed by steady growth to approximately 130 thousand units per month by late 2024. Atomoxetine increased from 1.5 to 2.5 thousand to 8–12 thousand units per month during the pandemic surge, with a continuous upward trend thereafter and multiple months in 2024 exceeding 14 thousand units. Clonidine, which is used in ADHD and other conditions, rose from 3.5 to 4.5 million to more than 12 million units per month during 2021 (threefold increase) before returning to baseline in the following years.

ADHD ‘core’ medications.

**FIGURE 2:**
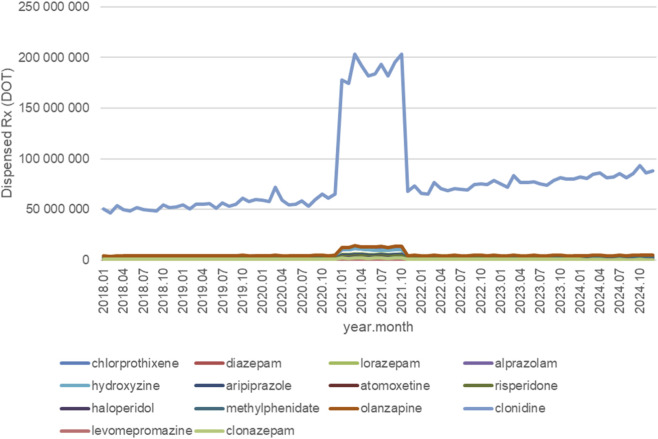
Neurodevelopmental and pervasive disorders drugs use in children and adolescents in Poland. Axis y: number of units, axis x: months and years of analysis.

Figure 2 shows monthly dispensing volumes for each analyzed active substance between January 2018 and December 2024. Most medications maintained relatively stable monthly dispensing volumes throughout the study period, with two notable exceptions: hydroxyzine and diazepam.

**FIGURE 3:**
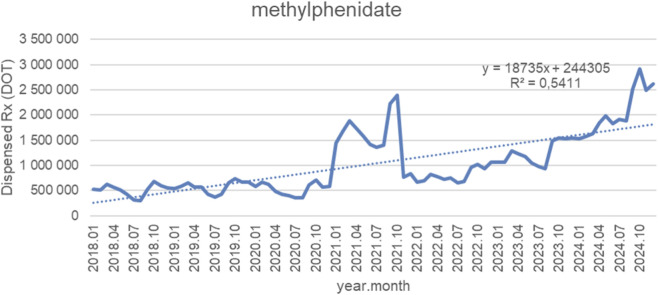
Methylphenidate use in children and adolescents in Poland. Axis y: number of units, axis x: months and years of analysis.

Figure 3 presents monthly dispensed days of therapy (DOT) for methylphenidate between January 2018 and October 2024, accompanied by a fitted linear trendline (y = 18 735x + 244 305; *R* ^2^ = 0.5411). Across the observation period, dispensed volumes showed a pronounced upward trajectory, with an average monthly increase of roughly 18,700 DOT. Between 2018 and late 2020, monthly values fluctuated widely—generally between 300,000 and 700,000 DOT—without clear long-term growth. A marked surge occurred in early 2021, with dispensing peaking near 2,500,000 DOT in mid-2021. This spike was followed by a sharp correction to pre-surge levels in late 2021 and early 2022. From mid-2022 onward, dispensing began to rise again, this time more steadily and persistently. The acceleration continued throughout 2023 and 2024, with volumes surpassing previous peaks and reaching historically highest levels of nearly 3,000,000 DOT by mid-2024. Although a brief dip followed in late 2024, the overall levels remained substantially above any pre-2023 period. The *R* ^2^ value of 0.5411 indicates that a linear model captures the long-term upward pattern reasonably well, although short-term deviations—particularly the pronounced pandemic-era spike and correction—introduce variability around the trendline.

**FIGURE 4:**
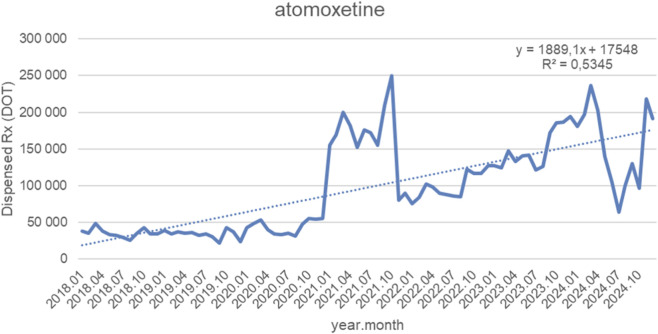
Atomoxetine use in children and adolescents in Poland. Axis y: number of units, axis x: months and years of analysis.

Figure 4 displays monthly dispensed days of therapy (DOT) for atomoxetine from January 2018 to September 2024, together with the fitted linear trendline (y = 1 889.1x + 17 548; *R* ^2^ = 0.5345). Over the study period, dispensing volumes followed a clear upward trend, increasing on average by approximately 1,900 DOT per month. From 2018 through mid-2020, monthly volumes remained relatively low and stable, fluctuating between 20,000 and 50,000 DOT, with no sustained growth. A substantial and abrupt rise began in early 2021, with dispensing rapidly climbing above 150,000 DOT and peaking at around 250,000 DOT in mid-2021. This surge was followed by a sharp correction in late 2021, when levels briefly returned below 100,000 DOT. Beginning in 2022, dispensing volumes resumed a steady upward trend, consistently surpassing pre-2021 levels. Throughout 2023 and 2024, atomoxetine use continued to expand, with multiple peaks between 180,000 and 230,000 DOT, despite occasional short-term drops—most notably a pronounced dip in mid-2024. By late 2024, volumes again rebounded above 180,000 DOT, indicating persistent long-term growth. The *R* ^2^ value of 0.5345 suggests that a linear model provides a reasonable representation of the overall increasing trend, although short-term volatility—particularly the 2021 surge and subsequent fluctuations—contributes to deviations around the trendline.

**FIGURE 5:**
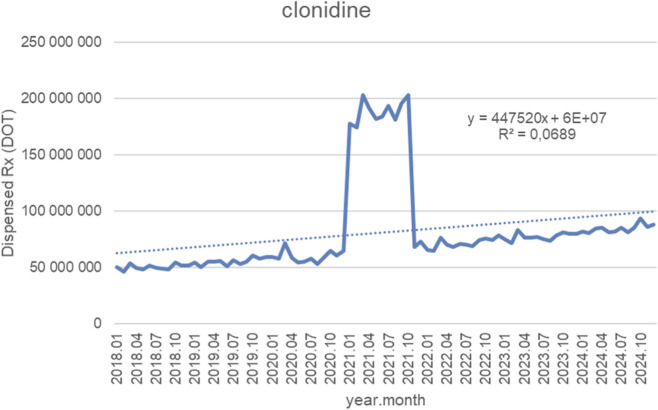
Clonidine use in children and adolescents in Poland axis y: number of units, axis x: months and years of analysis.

Figure 5 depicts monthly dispensed days of therapy (DOT) for clonidine from January 2018 through October 2024, alongside the fitted linear trendline (y = 447,520x + 6 × 10^7^; *R* ^2^ = 0.0689). Overall, dispensing volumes remained relatively stable during most of the observation period, fluctuating between approximately 45,000,000 and 70,000,000 DOT from 2018 to mid-2020, with only modest gradual increases. A dramatic and atypical spike emerged at the beginning of 2021, when monthly volumes surged abruptly to nearly 200,000,000 DOT and remained at this elevated level for several consecutive months. This temporary surge—far exceeding both preceding and subsequent values—was followed by a rapid return to baseline patterns by late 2021. From 2022 onward, dispensed volumes stabilized again around pre-spike levels, showing a slow upward drift but without further extreme deviations. Monthly volumes generally ranged from 70,000,000 to 90,000,000 DOT through 2023 and 2024, demonstrating moderate growth over time. The very low *R* ^2^ value (0.0689) indicates that a linear trendline explains only a small proportion of the overall variance. This is primarily due to the pronounced 2021 spike, which dominates the dataset and introduces substantial volatility, obscuring any underlying long-term linear trend in dispensing behavior.

### Antipsychotics

**FIGURE 6:**
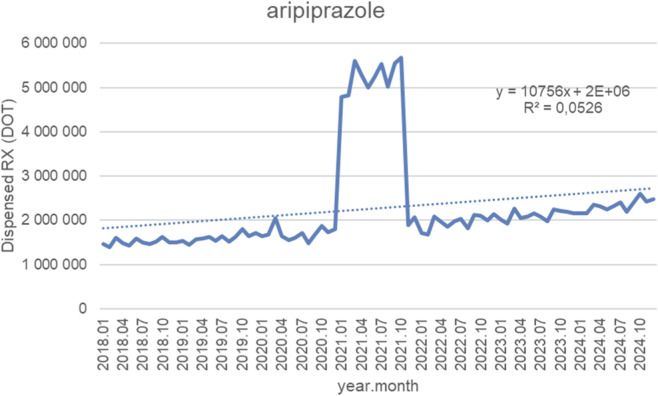
Aripiprazole use in children and adolescents in Poland. Axis y: number of units, axis x: months and years of analysis.

Figure 6 presents monthly dispensed days of therapy (DOT) for aripiprazole from January 2018 to September 2024, together with the fitted linear trendline (y = 10 756x + 2,000,000; *R* ^2^ = 0.0526). Throughout most of the period, dispensing volumes remained relatively stable, fluctuating between 1.4 million and 2.0 million DOT from 2018 to late 2020, with only gradual increases over time. A pronounced surge occurred at the start of 2021, when monthly dispensing rose sharply to approximately 5–5.5 million DOT and remained at this elevated level for several consecutive months. This short-lived spike was followed by a rapid return to baseline levels by early 2022, after which volumes stabilized again in the range of 1.8–2.4 million DOT. From 2022 through 2024, dispensing demonstrated a modest upward drift but no further extreme fluctuations. Despite this slow growth, the dataset is dominated by the abrupt 2021 spike, which produces substantial variability and weakens the explanatory power of the linear model. Correspondingly, the very low *R* ^2^ value (0.0526) indicates that the linear trendline captures only a small fraction of overall variation. This suggests that dispensing behavior for aripiprazole is largely characterized by stability with one exceptional, short-term deviation rather than by consistent linear growth over time.

**FIGURE 7:**
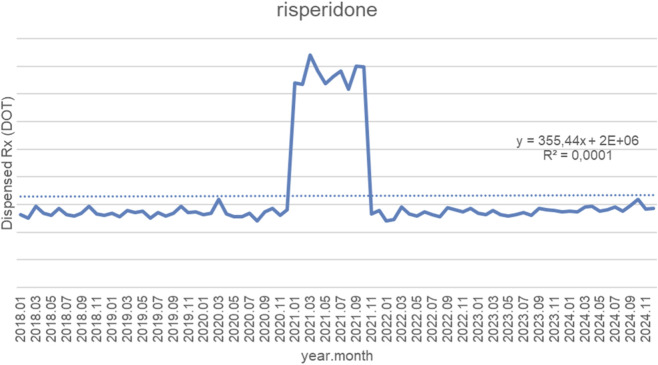
Risperidone use in children and adolescents in Poland. Axis y: number of units, axis x: months and years of analysis.

Figure 7 displays monthly dispensed days of therapy (DOT) for risperidone from January 2018 to September 2024, along with the fitted linear trendline (y = 355.44x + 2,000,000; *R* ^2^ = 0.0001). Throughout nearly the entire study period, monthly dispensing volumes remained remarkably stable, fluctuating within a narrow band of approximately 1.2–1.6 million DOT, with no meaningful long-term upward or downward movement. A single, pronounced deviation occurred in early 2021, when dispensing abruptly increased to around 3.5–4.0 million DOT for several consecutive months. This temporary spike was followed by a rapid return to baseline levels by late 2021. After this correction, dispensing patterns reverted to the earlier stable trend, remaining close to pre-spike levels for the remainder of 2022–2024. The extremely low *R* ^2^ value (0.0001) indicates that the linear trendline provides virtually no explanatory power for the observed variance. This is expected given the overwhelmingly flat pattern across most of the period, combined with one exceptional short-term spike that disrupts—but does not change—the underlying stable long-term dispensing behavior.

**FIGURE 8:**
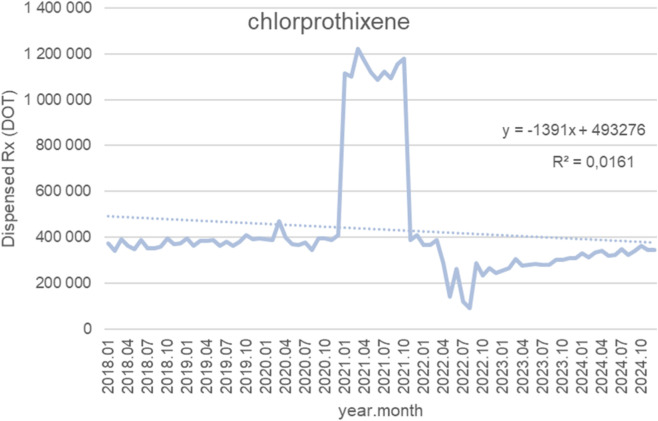
Chlorprothixene use in children and adolescents in Poland. Axis y: number of units, axis x: months and years of analysis.

Figure 8 presents monthly dispensed days of therapy (DOT) for chlorprothixene from January 2018 to October 2024, together with the fitted linear trendline (y = −1,391x + 493,276; *R* ^2^ = 0.0161). For most of the period, dispensing volumes were relatively stable, typically oscillating between 350,000 and 430,000 DOT from 2018 through 2020, with no clear long-term change. A substantial but short-lived spike occurred in early 2021, when monthly dispensing abruptly increased to approximately 1.1–1.2 million DOT and remained elevated for several months. This surge was followed by a rapid return to baseline levels in late 2021. Subsequently, in 2022, dispensing temporarily fell below historical norms, reaching a low of around 250,000 DOT before gradually recovering. From mid-2022 to 2024, monthly volumes trended upward again, stabilizing near 360,000–380,000 DOT by late 2024—like pre-spike levels. Although the trendline shows a slight negative slope, the extremely low *R* ^2^ (0.0161) indicates that a linear model explains almost none of the variability. This pattern reflects long-term stability interrupted only by a single, atypical spike and a brief post-spike decline, rather than any meaningful linear trend in utilization over time.

**FIGURE 9:**
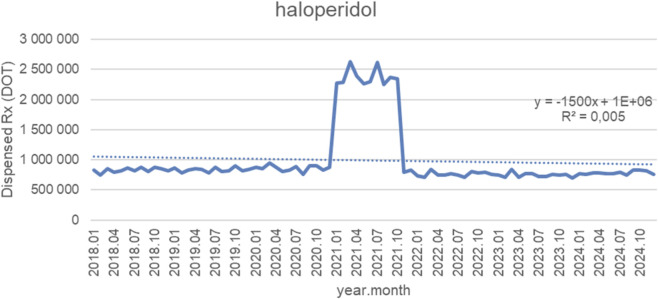
Haloperidol use in children and adolescents in Poland. Axis y: number of units, axis x: months and years of analysis.

Figure 9 presents monthly dispensed days of therapy (DOT) for haloperidol from January 2018 to October 2024, along with the fitted linear trendline (y = −1,500x + 1 × 10^6^; *R* ^2^ = 0.005). Across nearly the entire observation period, dispensing remained remarkably stable, with monthly volumes consistently fluctuating between approximately 800,000 and 1,050,000 DOT from 2018 through late 2020.

A marked but short-lived surge occurred in early 2021, when dispensing increased sharply to around 2.2–2.6 million DOT for several consecutive months. This temporary spike was followed by a rapid return to baseline levels by late 2021. After the correction, monthly volumes stabilized again within the typical pre-spike range, remaining around 800,000–900,000 DOT throughout 2022–2024.

Although the fitted trendline suggests a slight downward slope, the extremely low *R* ^2^ value (0.005) indicates that the linear model explains virtually none of the variance. The pattern is dominated by overall long-term stability interrupted only by one exceptional, short-duration spike, rather than by any meaningful linear change in utilization over time.

**FIGURE 10:**
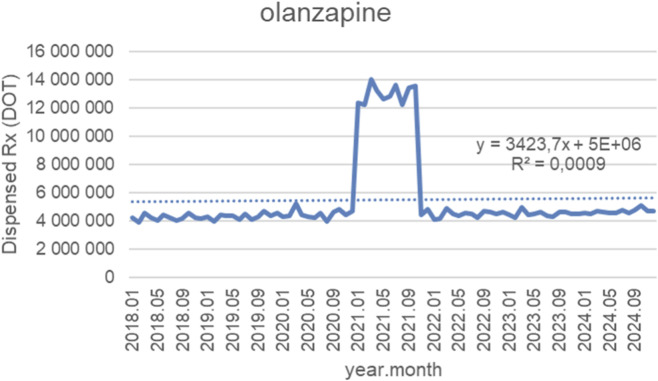
Olanzapine use in children and adolescents in Poland. Axis y: number of units, axis x: months and years of analysis.

Figure 10 illustrates monthly dispensed days of therapy (DOT) for olanzapine from January 2018 to October 2024, together with the fitted linear trendline (y = 3 423.7x + 5 × 10^6^; *R* ^2^ = 0.0009). For the majority of the study period, dispensing remained highly stable, with monthly volumes consistently ranging between approximately 4.5 and 6.0 million DOT from 2018 through 2020, showing minimal long-term variability. A notable but short-lived spike occurred in early 2021, when dispensing rose sharply to around 12–14 million DOT for several months. This surge was followed by a rapid return to baseline levels by late 2021. From 2022 onward, dispensing again stabilized at pre-spike levels, fluctuating narrowly around 5–5.5 million DOT through 2024, with no meaningful upward or downward trend. Although the fitted trendline suggests a slight positive slope, the extremely low *R* ^2^ value (0.0009) indicates that a linear model explains virtually none of the overall variance. This reflects the underlying pattern: long-term stability punctuated only by a single, exceptional and temporary utilization spike in 2021, rather than any systematic linear change over time.

**FIGURE 11:**
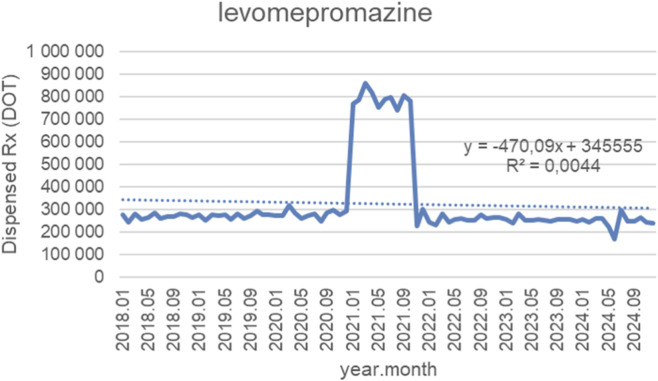
Levomepromazine use in children and adolescents in Poland. Axis y: number of units, axis x: months and years of analysis.

Figure 11 presents monthly dispensed days of therapy (DOT) for levomepromazine from January 2018 to October 2024, along with the fitted linear trendline (y = −470.09x + 345,555; *R* ^2^ = 0.0044). Throughout nearly the entire study period, dispensing remained highly stable, with monthly volumes consistently fluctuating between approximately 230,000 and 320,000 DOT from 2018 to 2020, showing no meaningful long-term upward or downward trend. A temporary and pronounced surge occurred in early 2021, when dispensing increased sharply to roughly 750,000–850,000 DOT over several months. This exceptional spike was followed by a rapid return to baseline levels by late 2021. From 2022 through 2024, dispensing once again stabilized within its historical range, fluctuating modestly between 230,000 and 300,000 DOT, with no signs of sustained growth or decline. Although the trendline indicates a slight negative slope, the extremely low *R* ^2^ value (0.0044) demonstrates that the linear model explains virtually none of the variation in the data. This reflects a pattern dominated by long-term stability with one short-lived utilization spike, rather than any underlying linear trend in levomepromazine dispensing over time.

### Sedating and anxiolytic agents

**FIGURE 12:**
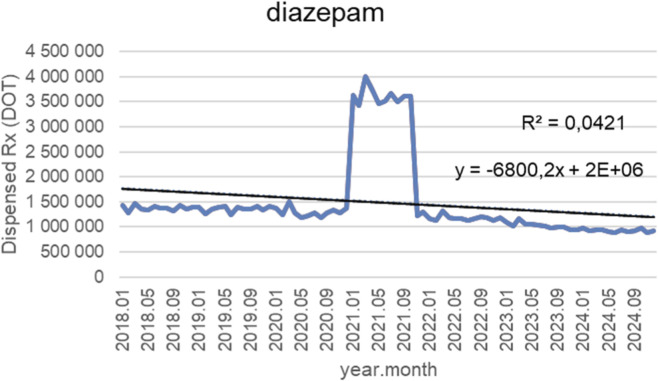
Diazepam use in children and adolescents in Poland. Axis y: number of units, axis x: months and years of analysis.

Figure 12 illustrates monthly dispensed days of therapy (DOT) for diazepam from January 2018 to October 2024, together with the fitted linear trendline (y = −6,800.2x + 2 × 10^6^; *R* ^2^ = 0.0421). For most of the study period, dispensing volumes remained relatively stable, typically ranging between 1.2 and 1.6 million DOT from 2018 through 2020, with only minor month-to-month variability. A sharp, short-lived spike occurred in early 2021, when monthly dispensing rose abruptly to approximately 3.54.0 million DOT and remained elevated for several months. This exceptional surge was followed by a rapid return to baseline levels by late 2021. From 2022 onward, dispensed volumes stabilized again but at slightly lower levels than before the spike, generally fluctuating between 1.0 and 1.3 million DOT through 2024. No sustained upward or downward shifts were observed outside of routine variability. Although the trendline indicates a modest downward slope, the low *R* ^2^ value (0.0421) suggests that a linear model explains only a small fraction of the overall variance. The overarching pattern reflects long-term stability with one prominent but temporary spike rather than any meaningful linear trend in utilization over time.

**FIGURE 13:**
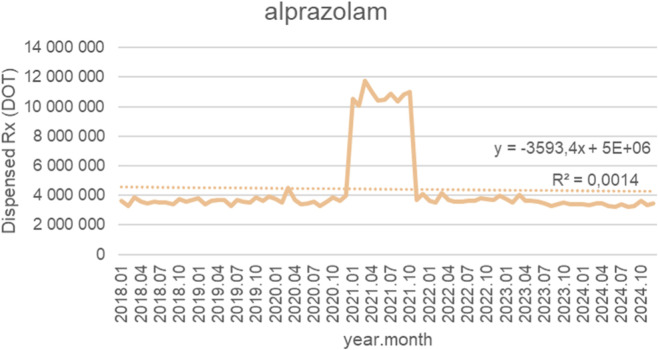
Alprazolam use in children and adolescents in Poland. Axis y: number of units, axis x: months and years of analysis.

Figure 13 depicts monthly dispensed days of therapy (DOT) for alprazolam from January 2018 to October 2024, together with a fitted linear trendline (y = −3,593.4x + 5 × 10^6^; *R* ^2^ = 0.0014). Across the pre-pandemic years (2018–2020), dispensing remained relatively stable, fluctuating between approximately 3.2 and 3.8 million DOT per month, with minimal long-term variation. In early 2021, a sharp and temporary surge occurred, with monthly dispensing rising dramatically to around 10–12 million DOT. This elevated utilization persisted for several months but was followed by a rapid return to baseline levels by late 2021. From 2022 through 2024, alprazolam dispensing stabilized again within the pre-2021 range, typically between 3.0 and 3.6 million DOT per month. No sustained upward or downward trend is apparent in the post-pandemic period. Despite the slightly negative slope of the trendline, the very low *R* ^2^ value (0.0014) indicates that the linear model explains virtually none of the observed variability. Overall, the pattern reflects long-term stability interrupted only by a single, short-lived utilization spike associated with the early pandemic period.

**FIGURE 14:**
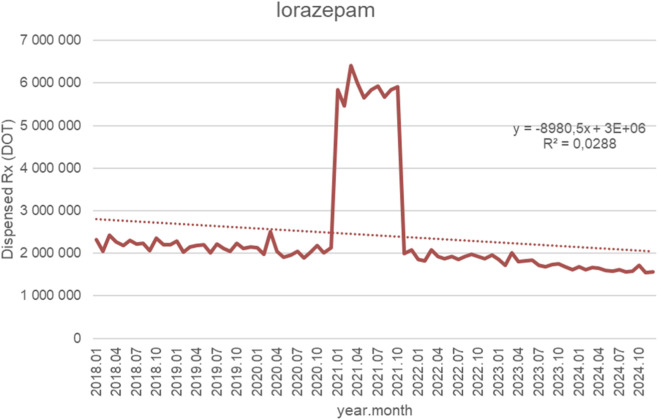
Lorazepam use in children and adolescents in Poland. Axis y: number of units, axis x: months and years of analysis.

Figure 14 illustrates monthly dispensed days of therapy (DOT) for lorazepam from January 2018 to October 2024, accompanied by the fitted linear trendline (y = −8,980.5x + 3 × 10^6^; *R* ^2^ = 0.0288). During the pre-2021 period (2018–2020), dispensing volumes remained relatively stable, typically fluctuating between 2.0 and 2.4 million DOT, with only minor month-to-month variability and no appreciable long-term trend. A sharp and temporary surge occurred at the beginning of 2021, when monthly dispensing increased dramatically to approximately 5.5–6.5 million DOT. This elevated utilization persisted for several months before returning rapidly to baseline levels in late 2021. From 2022 onwards, dispensing stabilized again but at slightly lower levels compared with the pre-spike period, generally ranging from 1.6 to 2.0 million DOT. A gradual downward drift is observable through 2022–2024, though short-term fluctuations remain limited. Despite the negative slope of the trendline, the low *R* ^2^ value (0.0288) indicates that the linear model explains only a small proportion of the overall variability. This reflects a utilization pattern characterized by long-term stability, interrupted only by a single pronounced and transient spike rather than any consistent linear trend over time.

**FIGURE 15:**
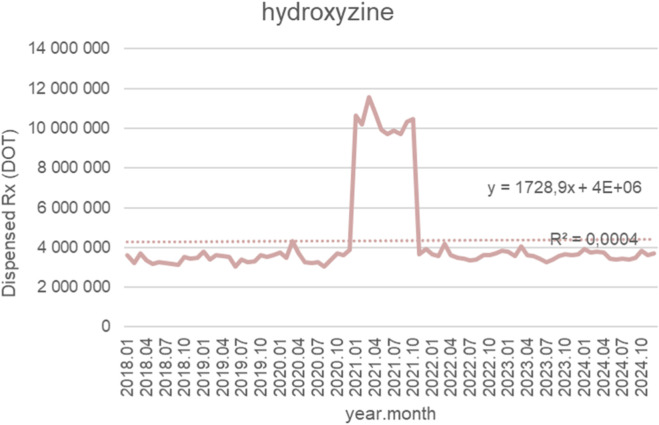
Hydroxyzine use in children and adolescents in Poland. Axis y: number of units, axis x: months and years of analysis.

Figure 15 shows monthly dispensed days of therapy (DOT) for hydroxyzine from January 2018 to October 2024, together with the fitted linear trendline (y = 1,728.9x + 4 × 10^6^; *R* ^2^ = 0.0004). For most of the observation period, dispensing remained highly stable, generally fluctuating between 3.0 and 4.0 million DOT from 2018 through 2020, with modest month-to-month variation and no meaningful long-term change. A sharp, temporary utilization spike occurred in early 2021, when monthly dispensing rose abruptly to approximately 10–12 million DOT and remained elevated for several months. This surge—far exceeding historical levels—was followed by a rapid return to baseline patterns in late 2021. From 2022 onward, hydroxyzine dispensing stabilized once again near pre-spike values, typically ranging between 3.0 and 3.6 million DOT. No sustained upward or downward trend is evident in the post-2021 period. Although the trendline indicates a very slight upward slope, the extremely low *R* ^2^ value (0.0004) demonstrates that the linear model explains virtually none of the variance in the data. This reflects a pattern characterized by long-term stability interrupted by a single, short-lived spike rather than any meaningful linear trend in utilization over time.

### Mathematical modeling

A modified Gompertz model was used to mathematically describe the drug’s unit accumulation. This model was used to mathematically describe cancer cell growth and to mathematically describe COVID-19 accumulation curves (Volpe, 2009; Bel and linger, 2008). This model has three parameters that correspond to the physical values of the phenomenon being described. Based on the model, predictions can be made that allow for obtaining a value for the maximum dose and enable the calculation of the time it will take this value. The modified Gompertz Model is presented by Equation 1.

$$\text{ym}\left(t\right)=\text{am}\cdot \exp\left[-\exp((\text{br}\cdot \;e/\text{am})\cdot \left(\text{ct}-t\right)+1)\right]$$ (1)

Where:

ym(t) – doses accumulation (drugs unit)

am–maximum doses (drugs unit)

br–doses rate (drugs unit/year)

ct–time of adaptation (year)

t–time (year)

e - basis of the natural logarithm (2.718)

**FIGURE 16:**
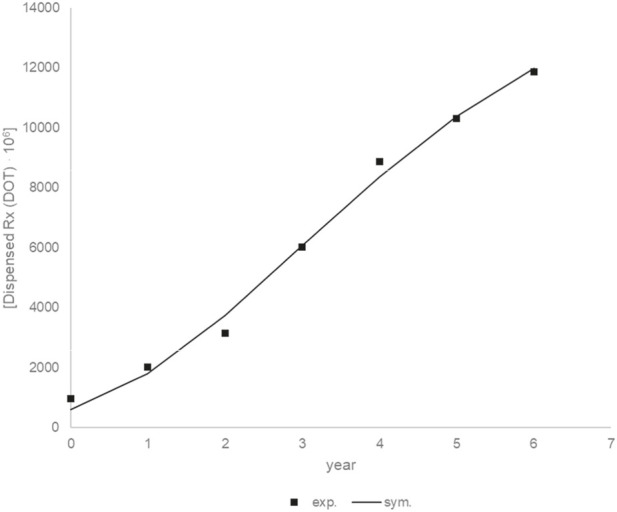
Experimental and simulation data for the drugs unit accumulation curve.

Figure 16 shows the experimental and simulation data for the dose accumulation curve. The fit of the experimental results to the model results is good (*R* ^2^ = 0.9931). The following values were obtained for the parameters: am_1_ = 15906,11 10^6^ (drugs unit), br_1_ = 2382,34 10^6^ (drugs unit/year), ct_1_ = 0.45 (year).

**FIGURE 17:**
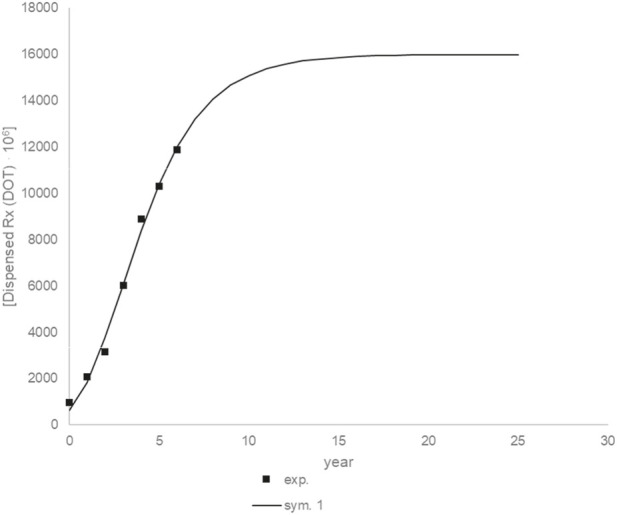
Experimental and simulation data for the drugs unit accumulation curve–prediction up to 25 years.

Figure 17 shows the prediction up to 25 years using modeling data. Drug units should continue to grow for about 10 years, after which they should stabilize. After 10 years, there should be an increase of about 5408,99 10^6^ drugs units. This value is calculated for a situation in which no other factors occur during this time, which is not the case, but it can provide information about the trend.

**FIGURE 18:**
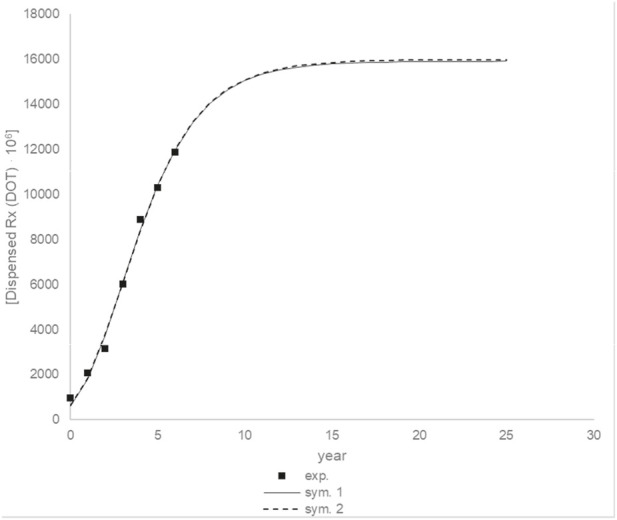
Comparison of simulations, sym. 1 and sym. 2 for different parameters.

Due to the uncertainty of the 2019 data, additional calculations were performed, which included determining constant parameters and conducting simulations with them. The following values were obtained for the parameters: am_2_ = 15875,00 10^6^ (drugs unit), br_2_ = 2372,45 10^6^ (drugs unit/year), ct_2_ = 0,44 (year), *R* ^2^ = 0,9929. Figure 18 shows comparison of simulations between different parameters. The simulation curves are almost identical.

## Discussion

The observed dispensing patterns reveal a distinct, synchronized surge in psychotropic medication use during 2021 across multiple therapeutic classes, followed by divergent post-pandemic trajectories. Although the dataset encompasses all clinical indications, these trends have important implications for treatment patterns in neurodevelopmental disorders (NDD), where several of the studied agents are widely prescribed. Because the dataset includes all-indication aggregated dispensing volumes and lacks patient-level diagnostic information, the interpretation of trends must remain cautious and cannot be directly linked to confirmed clinical changes in neurodevelopmental disorders. This limitation restricts the ability to infer whether the observed patterns reflect changes in diagnostic activity, symptom severity, or clinical management of NDD, and it also weakens comparability with studies based on diagnosis-linked claims data.

The spike in 2021 was most prominent for sedating and anxiolytic agents such as hydroxyzine, diazepam, alprazolam, and lorazepam, all of which experienced 2.5–3-fold increases over baseline volumes. This transient elevation aligns with international reports of increased sedative–hypnotic and benzodiazepine prescribing during the COVID-19 pandemic, attributed to heightened anxiety, insomnia, and behavioral dysregulation during lockdowns and school closures (Yanga et al., 2020; Taquet et al., 2021; Lee, 2020; Kaye et al., 2020). Post-peak, most of these medications reverted to or below baseline, reflecting either resolution of acute stressors or deliberate deprescribing initiatives in line with safe-use guidelines (Klimkiewicz et al., 2021).

Antipsychotics used in NDD including olanzapine, aripiprazole, risperidone, chlorprothixene, haloperidol, and levomepromazine demonstrated similarly sharp but short-lived increases, with volumes returning to pre-pandemic levels by late 2021. The uniformity of these peaks suggests that supply chain dynamics and precautionary stockpiling, in addition to clinical demand, may have contributed. Comparable surges have been documented in European markets for antipsychotics and mood stabilizers in the first year of the pandemic (Dell’Osso et al., 2015; Danielson et al., 2023).

When viewed alongside international findings, the Polish data offer several system level features that differentiate them from diagnostic claims-based studies, such as the Japanese interrupted time series analysis by Huang et al. (2025). That study was able to quantify diagnosis specific changes in psychotropic use during the pandemic, while our aggregated dataset does not permit indication linked modeling. However, the Polish results provide unique insights not captured in claims-based analyses. The unusually synchronized and sharp surge across sedatives, anxiolytics, and antipsychotics in early 2021 has not been reported with comparable magnitude in other national datasets and likely reflects Poland’s distinct combination of prolonged school closures, restricted access to non-pharmacological services, and broad reimbursement of psychotropic medications. These conditions created a system wide response that differs from patterns observed in East Asian or Western European settings, adding a novel perspective to the international literature on pandemic related prescribing dynamics.

A similar study, based on a national database, was conducted in France to analyze the use of psychotropic medications in children. It included a wide range of medications, including the following classes: N05A (antipsychotics), N05B (anxiolytics), N05C (hypnotics and sedatives), N06A (antidepressants), and N06B (psychostimulants), prescribed between 2018 and 2021. Similar to our study, a significant increase in prescriptions was observed in 2021, ranging from +20.7% to +689%, depending on the drug class examined. Comparisons between the COVID-19 pandemic and pre-pandemic periods revealed a significantly higher average reimbursement of psychotropic medications during the pandemic period for all classes except psychostimulants (Couturas et al., 2024). In the population of children aged 5–15 years in Ireland, a 32% increase in the number of prescribed psychotropic medications was also observed between 2017 and 2021 (Par et al., 2025). In a study by Amill-Rosario et al., almost a quarter of the nine million children aged 2–17 years used psychotropic medications during the 2019–2020 period, with the largest increase of nearly 40% recorded in April 2020 (Amill-Rosario et al., 2022). Before the COVID-19 pandemic, the estimated monthly prescription rate for psychotropic medications increased by an average of 0.4% per month. However, during the pandemic, this trend changed significantly, with the prescription rate increasing by up to 1.3% per month. An increase in prescription trends was observed for all classes of psychotropic medications, but this was particularly pronounced for anxiolytics, hypnotics, sedatives, and antidepressants. Prescription rates increased above expectations for all classes of psychotropic medications except psychostimulants (Valtuille et al., 2024).

In contrast, ADHD pharmacotherapies showed a markedly different trajectory. While methylphenidate and atomoxetine exhibited modest pandemic-related increases, their most notable feature was sustained growth through 2022–2024, surpassing pre-2020 levels. This mirrors global patterns of rising ADHD diagnosis rates and pharmacologic treatment in children and adults during and after the pandemic (Patel et al., 2020; Cortese and Coghill, 2018; Silczuk et al., 2025). Proposed drivers include increased clinical recognition due to remote learning challenges, improved care access *via* telemedicine, and heightened parental engagement in behavioral symptom reporting (Cucinotta and Vanelli, 2020). It should be emphasized that population-based studies in the United States report similar findings, indicating a sustained upward trend in stimulant prescribing after 2020 (Danielson et al., 2023).

Although clonidine is prescribed for multiple indications, including hypertension, its pandemic peak followed by reversion suggests that, unlike ADHD-specific agents, its use in behavioral disorders did not undergo long-term change.

From a public health perspective, these findings are of considerable importance. First, they indicate that in systemic crises such as the pandemic, there may be abrupt but transient changes in the use of certain psychotropic medications (e.g., benzodiazepines, antipsychotics), which subsequently return to baseline levels. Second, they highlight the emergence of structural changes that persist beyond the crisis period, as observed in the case of ADHD medications. Such lasting shifts may reflect changes in diagnostic practices, clinical management, and societal perceptions of neurodevelopmental disorders. Future research should focus on analyses of individual-level patient data, with careful differentiation of clinical indications, to better understand whether the observed increase in ADHD medication use corresponds to appropriate diagnosis and treatment or rather reflects overdiagnosis and the risk of medical overuse. It is also crucial to monitor the long-term safety of stimulant use in pediatric populations, particularly in the context of co-occurring neurodevelopmental and psychiatric disorders.

### Limitations of the study

This study has several important limitations that should be considered when interpreting its findings. First, the analysis was based on aggregated national reimbursement data from the IQVIA Pharmascope database, which does not include patient-level information such as age, sex, clinical diagnosis, treatment duration, dosage, or therapeutic indication. Consequently, the results reflect overall dispensing trends rather than confirmed medication use in children with specific neurodevelopmental disorders. Second, the dataset covers only reimbursed outpatient prescriptions, excluding non-reimbursed drugs, hospital use, and private purchases, which may have influenced total consumption estimates. Third, the study design was descriptive and retrospective, relying primarily on visual and percentage-based comparisons between time periods without formal inferential testing (e.g., interrupted time-series or regression modeling). Therefore, observed changes cannot be causally attributed to the COVID-19 pandemic or specific policy shifts. Fourth, the study did not control potential confounding factors such as changes in diagnostic criteria, prescription regulations, healthcare accessibility, or socioeconomic conditions. Finally, the possibility of stockpiling behavior during lockdowns and temporary disruptions in drug supply may have artificially inflated dispensing volumes in 2021. Additionally, because the dataset consists of aggregated national monthly reimbursement volumes without patient-level diagnostic information, advanced time-series methods (e.g., interrupted time-series analysis with seasonality adjustment) could not be reliably applied. In addition, the lack of indication-specific information in the reimbursement dataset introduces an additional methodological constraint that directly affects the interpretability of the findings. Because dispensed units cannot be linked to confirmed diagnoses such as ADHD, ASD, or intellectual disability, the trends presented here should not be interpreted as diagnosis-specific prescribing patterns. This limitation also weakens comparability with international studies that use claims data linked to diagnostic codes or patient-level clinical characteristics and therefore permit more rigorous causal inference or formal time-series analyses. As a result, our conclusions are intentionally conservative, focusing on descriptive patterns rather than etiological explanations or changes in clinical management. Finally, the Gompertz modeling presented in this study is exploratory; its parameters are not externally validated, and the long-term prediction should be interpreted with caution due to the absence of sensitivity analyses. Despite these limitations, the national coverage and multi-year perspective provide valuable insights into large-scale prescribing dynamics and long-term trends in psychotropic medication use among pediatric populations in Poland.

### Future directions

Future research should build upon these findings by employing individual-level pharmacoepidemiologic data that link prescription records with diagnostic information, age, sex, and treatment outcomes. Such linkage would enable differentiation between appropriate and potentially excessive prescribing, particularly for ADHD medications, which showed sustained post-pandemic growth. Further studies should also apply inferential statistical approaches, such as interrupted time-series analyses or segmented regression models, to quantify the magnitude and duration of pandemic-related effects and to test for statistical significance across time periods. It is also important to investigate regional and socioeconomic disparities in psychotropic drug use, exploring how access to psychiatric and psychological care, telemedicine availability, and healthcare infrastructure influenced prescribing behavior. Longitudinal cohort studies following children and adolescents treated with stimulants or antipsychotics could assess long-term safety, adherence, and functional outcomes. Additionally, future work should incorporate qualitative and behavioral research to understand clinician decision-making, parental attitudes, and the role of educational systems in shaping treatment demand. Finally, monitoring policy changes, such as updates in reimbursement criteria or clinical guidelines, will be essential to determine whether the pandemic has triggered a lasting structural shift in pediatric neurodevelopmental pharmacotherapy or a transient adaptation to crisis conditions.

## Conclusion

Across all therapeutic categories examined, a pronounced and synchronized spike in drug dispensing occurred in 2021, consistent with a pandemic-related surge (Figure 16). That would make patterns for ADHD drugs and lower-volume antipsychotics clearer. This was observed for sedatives, anxiolytics, antipsychotics, and ADHD medications, although the magnitude and post-peak trajectories differed between classes. High-volume agents such as olanzapine, clonidine, hydroxyzine, and diazepam showed absolute increases reaching several-fold above baseline, while lower-volume antipsychotics and benzodiazepines displayed similarly marked relative rises. Due to huge volumes of olanzapine/clonidine units, on Figure 17 there is a comparison by every drug without them, so smaller-volume drugs are easier to compare. For most sedatives, anxiolytics, and antipsychotics, the elevated dispensing rates reverted to or below pre-pandemic levels by late 2021, with gradual declines in subsequent years. In contrast, ADHD core pharmacological therapies (methylphenidate, atomoxetine) showed sustained post-pandemic growth, exceeding pre-2020 levels throughout 2022–2024.

## Data Availability

The original contributions presented in the study are included in the article/supplementary material, further inquiries can be directed to the corresponding author.
